# Charlson comorbidity index has no incremental value for mortality risk prediction in nursing home residents with COVID-19 disease

**DOI:** 10.1186/s12877-025-05721-2

**Published:** 2025-01-30

**Authors:** Anum Zahra, Maarten van Smeden, Petra J. M. Elders, Jan Festen, Jacobijn Gussekloo, Karlijn J. Joling, Anouk van Loon, Kim Luijken, René J. F. Melis, Simon P. Mooijaart, Karel G. M. Moons, Geeske Peeters, Harmke A. Polinder-Bos, Fenne Wouters, Anne de Hond

**Affiliations:** 1 Julius Center for Health Sciences and Primary Care, University Medical Center Utrecht, Utrecht University, Utrecht, The Netherlands; 2 Department of General Practice & Elderly Care Medicine, Amsterdam Public Health Research Institute, VU University Medical Centre, Amsterdam, The Netherlands; 3 KBO-PCOB, Etten-Leur, The Netherlands; 4 Department of Internal Medicine, Section of Geriatrics and Gerontology, Leiden University Medical Center, Leiden, The Netherlands; 5 LUMC Center for Medicine for Older People, Leiden University Medical Center, Leiden, The Netherlands; 6 Department of Public Health and Primary Care, Leiden University Medical Center, Leiden, The Netherlands; 7 Department of Medicine for Older People, Amsterdam UMC, Location Vrije Universiteit Amsterdam, De Boelelaan 1117, Amsterdam, The Netherlands; 8 Aging & Later Life, Amsterdam Public Health Research Institute, Amsterdam, The Netherlands; 9 Department of Geriatric Medicine, Radboud University Medical Center, Nijmegen, The Netherlands; 10 Division of Geriatric Medicine, Department of Internal Medicine, Erasmus MC, University Medical Center Rotterdam, Rotterdam, The Netherlands

**Keywords:** Charlson comorbidity index, Frailty, Vulnerability, COVID‐19, Older people, Prognosis research, Nursing home population

## Abstract

**Background:**

During the COVID-19 pandemic, nursing home (NH) residents faced the highest risk of severe COVID-19 disease and mortality. Due to their frailty status, comorbidity burden can serve as a useful predictive indicator of vulnerability in this frail population. However, the prognostic value of these cumulative comorbidity scores like the Charlson comorbidity index (CCI) remained unclear in this population. We evaluated the incremental predictive value of the CCI for predicting 28-day mortality in NH residents with COVID-19, compared to prediction using age and sex only.

**Methods:**

We included older individuals of ≥ 70 years of age in a large retrospective observational cohort across NHs in the Netherlands. Individuals with PCR-confirmed COVID-19 diagnosis from 1 March 2020 to 31 December 2021 were included. The CCI score was computed by searching for the comorbidities recorded in the electronic patient records. All-cause mortality within 28 days was predicted using logistic regression based on age and sex only (base model) and by adding the CCI to the base model (CCI model). The predictive performance of the base model and the CCI model were compared visually by the distribution of predicted risks and area under the receiver operator characteristic curve (AUROC), scaled Brier score, and calibration slope.

**Results:**

A total of 4318 older NH residents were included in this study with a median age of 88 years [IQR: 83–93] and a median CCI score of 6 [IQR: 5–7]. 1357 (31%) residents died within 28 days after COVID-19 diagnosis. The base model, with age and sex as predictors, had an AUROC of 0.61 (CI: 0.60 to 0.63), a scaled brier score of 0.03 (CI: 0.02 to 0.04), and a calibration slope of 0.97 (CI: 0.83 to 1.13). The addition of CCI did not improve these predictive performance measures.

**Conclusion:**

The addition of the CCI as a vulnerability indicator did not improve short-term mortality prediction in NH residents. Similar (high) age and number of comorbidities in the NH population could reduce the effectiveness of these predictors, emphasizing the need for other population-specific predictors that can be utilized in the frail NH residents.

**Graphical Abstract:**

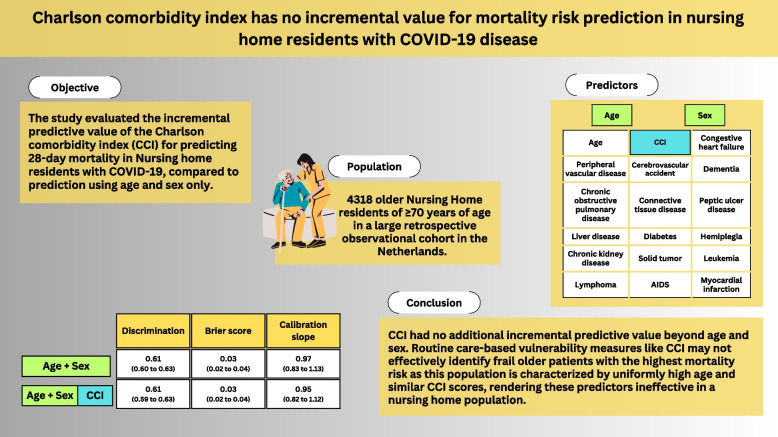

**Supplementary Information:**

The online version contains supplementary material available at 10.1186/s12877-025-05721-2.

## What this study adds

- Vulnerability of individuals, such as older people with multiple comorbidities, is thought to have played an important role in the exceptionally high mortality rates during the COVID-19 pandemic. Still, the impact of comorbidity indices in mortality risk prediction remains unknown.
- We assessed the incremental predictive value of the Charlson comorbidity index in addition to age and sex for 28-day mortality in a nursing home population ≥ 70 years of age with COVID-19.
- We found no evidence for incremental value of the Charlson comorbidity index extracted from nursing home records, as an indicator of vulnerability, in predicting mortality risk after COVID-19 diagnosis in addition to age and sex.
- Routine care-based vulnerability measures might not effectively identify frail older patients with the highest mortality risk. This population is characterized by uniformly high age and similar Charlson comorbidity index scores, possibly rendering these predictors ineffective in a nursing home population.
- Further research is needed to investigate other older population-relevant predictors reflecting functional (in)dependence status when assessing prognosis in the nursing home populations.

## Background

The COVID-19 pandemic caused significant loss of life, especially among older people residing in nursing homes (NH). Mortality rates in these settings were notably high [1–3] due to decreased (physical) resilience caused by decreased immunity and the presence of multiple comorbidities [4]. The impact of comorbidities on modifying the severity and outcomes of COVID-19 has been recognized since the beginning of the pandemic [5]. In the NH settings, the residents are predominantly frail, highlighting the need for older population-specific predictors like cumulative comorbidity scores that reflect vulnerability [6]. However, the role of comorbidity indices like the Charlson comorbidity index (CCI) [7], as an indicator of vulnerability for prognostication in older individuals in the NH settings, has not been studied so far.

CCI is a widely used, validated comorbidity score for estimating the risk of death from comorbid diseases in different healthcare settings and clinical conditions [8]. Multiple studies including a systematic review found CCI to be associated with adverse health outcomes (mechanical ventilation or critical care) and mortality in the (older) COVID-19 population [9, 10]. Additionally, most prediction models developed during the COVID-19 pandemic performed poorly in the older population (including frail NH residents) [11]. Previous studies have shown that age and sex are important predictive factors for COVID-19 mortality, with older men having the highest risk [3, 12, 13].

Based on these findings and the availability of information about comorbidities in NH residents’ health records, the present study aimed to evaluate the added value of CCI for predicting 28-day mortality after contracting COVID-19 disease beyond age and sex. Understanding the predictive role of NH-relevant vulnerability measures in COVID-19 prognosis is essential, as well as gaining insights into the demonstrated resilience of older individuals due to an acute illness. Moreover, this knowledge can provide valuable insights into developing accurate prediction models specifically intended for use in the NH population.

## Methods

The study was reported according to the transparent reporting of a multivariable prediction model for individual prognosis or diagnosis (TRIPOD + AI; www.tripod-statement.org) guidelines [14, 15] for prediction model development (Supplement 7) with sample size calculations (Supplement 2).

### Participants

The study population consisted of COVID-19 patients aged 70 years or older. Data were collected using COVID-19 registration forms integrated with the electronic health record of the NH residents containing information about age, sex at birth, mortality, date of death, and comorbidities [3]. NH residents with PCR-confirmed COVID-19 disease from 1 March 2020 to 31 December 2021 were included in the study. Only the first COVID-19 infection of the patients in this cohort was used in this study. Missing information of participants without comorbidities as listed in the CCI or with unknown mortality status during the follow-up period were excluded from the analysis.

### Charlson comorbidity index measurements

The CCI is a weighted index that includes 16 medical conditions including age [16]. Each condition is assigned a weight from 1 to 6 (Supplement 1). CCI is not routinely registered for NH residents and therefore comorbidities were extracted from the medical histories section of the electronic health records by a free text search for specified medical conditions using MATLAB [17]. Information about disease severity for certain comorbidities (liver disease and diabetes) could not be extracted from NH medical histories. Free text search terms per medical condition (supplemented with ICD-10 codes) were formulated in collaboration with a geriatric clinician and NH data experts as detailed in Supplement 1.

### Statistical analysis

Baseline characteristics were described separately for patients who died within 28 days after contracting COVID-19 and those who survived. Continuous variables were summarized using median and interquartile range, while categorical variables were described using counts with percentages. Univariable associations between mortality and baseline characteristics were analysed using the chi-square test for binary variables (fisher exact test for leukaemia and mild liver disease) and Pearson correlation for continuous variables. The incremental value of CCI was assessed using two models: a base model with age and sex as the only predictors and a CCI model containing age, sex and CCI scores. The outcome was the cumulative risk of 28-day mortality. Both models included age, sex, and the interaction between sex and age. Age and CCI were modelled as a restricted cubic spline function with 4 knots on the percentiles 0.05, 0.35, 0.65, and 0.95, to allow for a non-linear relation between these continuous predictors and mortality. We modelled age and the CCI as a restricted cubic spline to avoid loss of prognostic information caused by approaches like the categorization of these variables in two or more categories, or assuming linearity on a continuous level.

Each of the two models was estimated using penalized logistic regression (Firth’s regression). We obtained the apparent predictive performance and optimism-corrected performance using bootstrapping for each model. The incremental predictive value was evaluated by comparing the area under the receiver operator curve (AUROC) (including DeLong test to assess difference in the AUCs of the two models), scaled Brier score, calibration slope. The predictive performance of the base model and the CCI model were compared visually using density plots. Density plots show the distribution of predicted risks on a scale from 0 (0% risk of dying within 28 days) to 1 (100% risk of dying within 28 days). Density plots were generated separately for those with and without the outcome of 28-day mortality. A significant amount of overlap between the plots for the survivor and deceased groups indicates that the model finds it difficult to separate the two (this will translate in a low AUC). When the plots only have a little overlap, the model is successful at separating the groups (this will result in a high AUC). Changes in spread and separation in predicted risks between those who died or survived in the CCI model, compared to the base model, could indicate an improvement in predictive performance. Confidence intervals (95% CI) for the AUROC were calculated by the Delong variance estimation method and for the scaled Brier score a bootstrap sampling distribution from 1000 bootstrap samples was used. All analyses were performed in R 4.2.2, using the R packages logistf, rms and pROC [18].

### Sensitivity analysis

The incremental value of CCI was assessed separately in the years 2020 and 2021 by redeveloping the base and CCI models, as these periods reflected different COVID-19 mortality rates in the NH population. The incremental predictive value for the sensitivity analysis was evaluated by comparing the area under the ROC curve (AUROC), scaled Brier score, and calibration slope.

## Results

**Fig. 1:**
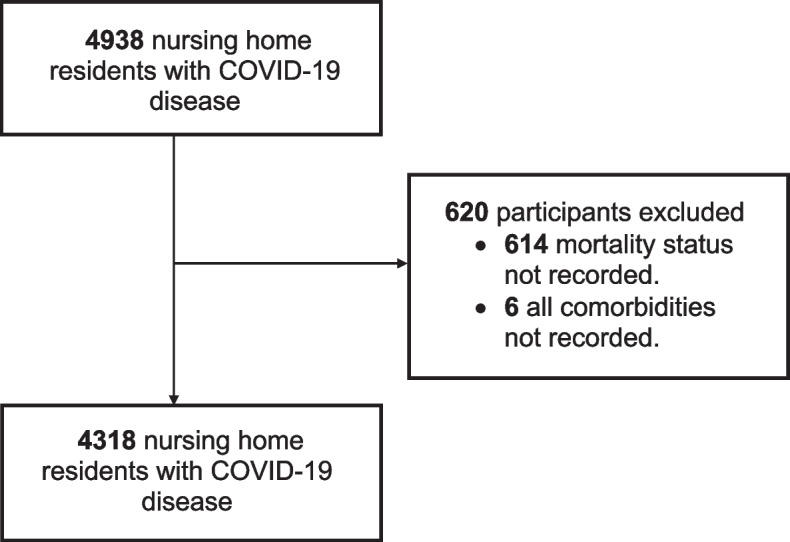
Flowchart for inclusion of older individuals in the nursing home cohort

We included 4318 older COVID-19 patients in this study (Fig. 1) including 2447 in chronic psychogeriatric care, 1030 in chronic somatic care, and 841 in revalidation or short-term care (details in Supplement 6). Of the study participants, 1,526 (35%) were male and had a median age of 88 years (IQR: 83–93). The median CCI score was 6 (IQR: 5–7), with a range from 3 to 18. In total, 31% (1357 individuals) of the study population died within 28 days. Dementia was the most frequent comorbidity (67%), followed by cancer (25%), diabetes (22%), chronic kidney disease (17%), and chronic heart failure (17%). The median age, percentage of males, and percentage of chronic kidney disease were higher in individuals who died than in individuals who survived after 28 days. Notably, the CCI scores were the same on average for deceased and surviving residents. Detailed characteristics of the study population can be found in Table 1. The study excluded 620 individuals (6 individuals without comorbidities registration, and 614 individuals for their missing mortality status).

**Table 1:** Characteristics of study participants stratified on mortality status

Characteristics		All participants (*n* = 4318)	Survivors (*n* = 2961)	28-day mortality (*n* = 1357)	*p*-value
**Age (years), median [IQR]**		88 [83–93]	87 [82–93]	89 [84–94]	< 0.001
**Male, n (%)**		1526 (35)	931 (31)	595 (44)	< 0.001
**Mortality, n (%)**		1357 (31)	-	-	-
**Charlson comorbidity index, median [IQR]**		6 [5–7]	6 [5–7]	6 [5–7]	0.012
**Dementia, n (%)**		2881 (67)	1965 (66)	916 (68)	0.482
**COPD, n (%)**		276 (6)	190 (6)	86 (6)	0.975
**Diabetes, n (%)**	**Uncomplicated**	895 (21)	617 (21)	278 (20)	0.823
	**End-organ damage**	43 (1)	27 (1)	16 (1)	0.512
**Chronic kidney disease, n (%)**		722 (17)	451 (15)	271 (20)	< 0.001
**Liver disease, n (%)**	**Mild**	8 (< 1)	7 (< 1)	1 (< 1)	0.449
	**Severe**	60 (1)	42 (1)	18 (1)	0.921
**Myocardial infarction, n (%)**		415 (10)	281 (10)	134 (10)	0.732
**Chronic heart failure, n (%)**		717 (17)	466 (16)	251 (19)	0.027
**Peripheral vascular disease, n (%)**		181 (4)	125 (4)	56 (4)	0.950
**Cerebrovascular accident, n (%)**		566 (13)	376 (13)	190 (14)	0.259
**Connective tissue disease, n (%)**		619 (14)	478 (16)	141 (10)	< 0.001
**Peptic ulcer disease, n (%)**		86 (2)	56 (2)	30 (2)	0.562
**Paralysis, n (%)**		12 (< 1)	7 (< 1)	5 (< 1)	0.650
**Solid tumor, n (%)**	**Localized**	972 (23)	676 (23)	296 (22)	0.482
	**Malignant**	87 (2)	60 (2)	27 (2)	1
**Leukemia, n (%)**		15 (< 1)	9 (< 1)	6 (< 1)	0.578
**Lymphoma, n (%)**		22 (1)	16 (1)	6 (< 1)	0.849

*p*-value of univariable association obtained using chi-square test for binary variables (fisher exact test for leukaemia and mild liver disease) and Pearson correlation for continuous variables

### Incremental predictive value

**Fig. 2:**
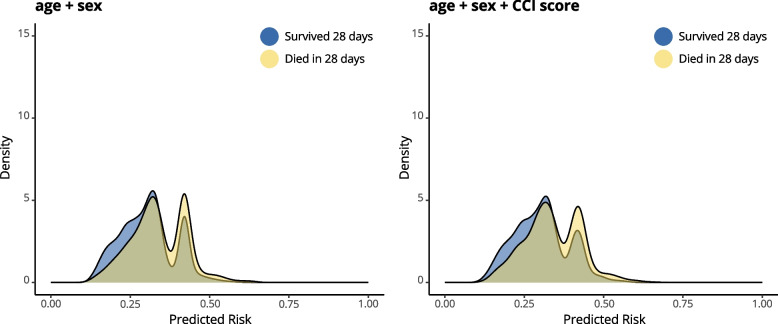
Distributions of predicted risks by penalized models stratified by outcome. Plot A shows the 28-day mortality risks as predicted by the base model, whereas plot B shows the risks predicted for the CCI model (both models showed very similar distribution). For each plot, the distribution of predicted risks for patients who survived 28 days is shown in blue, and the distribution of predicted risks for patients who died within 28 days is shown in yellow

The density plots showed that the predicted 28-day mortality risk ranged between 13 to 64% for the base model. Adding the CCI to the base model led to a similar spread of the distribution of predicted risks (range: 11% to 66%) (Supplement 3). No improvement in the (considerable) overlap in predicted risks of survivors and individuals who died within 28 days after addition of CCI to the base model indicated no incremental value of CCI (Fig. 2). Using penalized logistic regression, the CCI model showed minimal change in predictive performance, with no significant difference in AUROC between the two models (DeLong test p-value = 1), as well as similar calibration slope and scaled Brier score (detailed results in Supplement 3). The base model had an optimism-corrected predictive performance of AUROC 0.61 (CI: 0.59 to 0.63), scaled brier score 0.03 (CI: 0.01 to 0.06) and calibration slope 0.96 (CI: 0.82 to 1.15). This indicated that the base model displayed the same discrimination and brier scores as the CCI model (Table 2). Both models showed slight extremes in the distribution of estimated risks compared to predicted risks, with a calibration slope of less than one.

**Table 2:** Results of optimism corrected model performance using bootstrapping (*n* = 1000)

	**Base model** (age + sex + age*sex)	**CCI model** **Base model + CCI**
**AUROC, (95% CI)**	0.61 (CI: 0.60 to 0.63)	0.61 (CI: 0.59 to 0.63)
**Scaled Brier score, (95% CI)**	0.03 (0.02 to 0.04)	0.03 (0.02 to 0.04)
**Calibration slope, (95% CI)**	0.97 (0.83 to 1.13)	0.95 (0.82 to 1.12)

*CCI* Charlson comorbidity index, *AUROC* area under receiver operator curve, *CI* confidence interval

### Sensitivity analysis

The mortality fraction in year 2020 was higher (41%) than in year 2021 (25%). In line with the results of the primary analysis, CCI had no incremental predictive value in the separate analyses for the years 2020 and 2021 (Supplement 4 and 5).

## Discussion

This study evaluated the incremental value of CCI for 28-day mortality in frail NH residents after COVID-19 disease as a potentially useful predictive score for this setting. The study found a high 28-day mortality fraction in the NH population (32%). The addition of CCI score to reflect a patient’s vulnerability status did not improve the mortality risk prediction in this population compared to a base model with age and sex only with AUROC: 0.61 (CI: 0.60 to 0.63), brier score: 0.03 (CI: 0.02 to 0.04), calibration slope: 0.97 (CI: 0.83 to 1.13).

We could not identify studies examining the incremental value of CCI for COVID-19 mortality specifically in NH residents however a similar study investigating the incremental value of frailty and comorbidity indicators, including CCI in older COVID-19 individuals (primary care setting) reported an AUROC of 0.69 using penalized models with age and sex for the prediction of 28-day mortality [19]. These results align with our findings, showing the limited predictive value of CCI incremental beyond age and sex. Notably, age and sex alone also displayed low predictive performance in the older frail NH population (AUROC: 0.61) in the current study. Moreover, an external validation study GAL-COVID-19 model [20], including age, sex and 7 comorbidities as predictors, showed poor predictive performance for 28-day mortality prediction in the NH population [11]. Two meta-analyses and additional studies on early pandemic hospitalized COVID-19 patients and long-term care residents reported an association between CCI (or multiple comorbidities) and poor prognosis, where estimates from some individual studies were adjusted for age or/and sex [9, 21–25]. On the other hand, a recent retrospective cohort study demonstrated that pre-existing comorbidities, although highly prevalent in the older population, contributed minimally to in-hospital mortality when controlled for age and sex [12].

We may also look beyond COVID-19 disease and compare our results to the prediction literature for other acute infections, such as pneumonia or influenza. Consistent with our findings, a large population-based study on patients hospitalized for pneumonia found that routine care-based vulnerability measures (like the CCI) had very limited incremental predictive value for mortality besides age and sex [26]. Nevertheless, more research is needed to ascertain whether the limited predictive value observed is specific to the comorbidity indices used in this study or if other older population-specific predictors would also perform poorly in predicting short-term mortality after acute infections in NH residents. Since the values for age, comorbidities and frailty scores within this population remain high, the predictive value of these predictors is reduced. This emphasises the need for investigation of other older population-specific predictors, like indicators reflecting functional (in)dependence status (such as frailty scores) and age-independent predictors [27] in the frail NH population [11].

This study included a large cohort of NH residents that represents more than half of the NH in the Netherlands. Further strengths of this study include robust modelling with non-linear terms for continuous predictors (both age and CCI), which is better suited for assessing risk in older populations, as well as the use of multiple metrics to evaluate incremental predictive value.

There are also several limitations in this study. CCI was calculated by the open text field for medical history in the NH electronic health records. However, due to the use of NH patient files, CCI values may have been influenced by unregistered diagnoses and comorbidities, which may have led to an underestimation of the CCI and thereby underestimation of its predictive value if missing comorbidities affect patients with higher scores the most. Additionally, there is also an incomplete measurement of the CCI score (information about disease severity) was not extractable from NH patient files, which may have further underestimated the predictive value of the CCI. Another limitation of the study is the lack of further sensitivity analysis on the incremental value differences among NH residents based on their level of dependence (inadequately recorded with many missing values) or type of care received (Supplement 6). These factors may influence the incremental value and should be explored in future studies.

Lastly, the current study could not account for COVID-19 viral strains, potentially limiting the generalization of our results to current COVID-19 patients. However, our sensitivity analysis showed no differences in predictive performance in the years 2020 and 2021, suggesting that variations in viral strains did not significantly influence our findings. Factors relating to COVID-19 prevention, nutritional status [28], level of dependence or functional status [29], and patient management like COVID-19 vaccinations [30], social distancing policies [31] or treatment changes were not included in our study even though they could potentially influence the outcome and hence the prognostic value of the measured comorbidities.

Our study findings indicate that in NH residents, the CCI score as a comorbidity-based vulnerability score did not provide additional predictive information for 28-day mortality risk beyond what is already captured by age and sex. Future research is needed to investigate the generalizability of our results to other acute infectious diseases and other vulnerability measures like comorbidity scores specific to the older population such as the Cumulative Illness Rating Scale-Geriatric [32]. An interesting aspect would be to evaluate whether specific comorbidities [5], overall multiple long-term conditions [33] better represent vulnerability status in the older frail populations. This would provide useful insight for future pandemic preparedness and understanding resilience factors against acute illnesses, especially in older frail populations.

## Conclusion

When predicting 28-day mortality in older individuals presenting with COVID-19 in the NH setting, this study found that age and sex were (weak) predictive factors while CCI had no additional incremental predictive value. Therefore, caution should be exercised when using comorbidity scores like CCI as a vulnerability indicator to predict mortality in NH residents with COVID-19. Future studies should evaluate whether other more comprehensive predictors could effectively and rapidly identify (future pandemic) older frail individuals with an acute infection who are at high risk of poor outcomes.

## Supplementary Information

Below is the link to the electronic supplementary material.

Supplementary Material 1

## Data Availability

The data that support the findings of this study are available from “Leren van Data” but restrictions apply to the availability of these data, which were used under license for the current study, and so are not publicly available. Data are however available from the authors upon reasonable request and with permission of “Leren van Data”.

## Abbreviations

AUROC: Area Under the Receiver Operating Characteristic curve
CCI: Charlson Comorbidity Index
COOP: COVID Outcomes in Older People
EU GDPR: European Union General Data Protection Regulation
NH: Nursing Home
PCR: Polymerase Chain Reaction
TRIPOD: Transparent Reporting of a multivariable prediction model for Individual Prognosis Or Diagnosis
