# Supplementary material for: Charlson comorbidity index has no incremental value for mortality risk prediction in nursing home residents with COVID-19 disease
Source: BMC Geriatr. 2025 Jan 30;25:67. doi: 10.1186/s12877-025-05721-2 (PMC11780814; doi:10.1186/s12877-025-05721-2)
Supplement: Supplementary file 1 — Supplementary Material 1 [file 12877_2025_5721_MOESM1_ESM.pdf]

Supplementary materials

Charlson comorbidity index has no incremental value for mortality risk prediction in nursing home residents with COVID-19 disease

Anum Zahra, Maarten van Smeden, Petra J.M. Elders, Jan Festen, Jacobijn Gussekloo, Karlijn J Joling, Anouk van Loon, Kim Luijken, René J.F. Melis, Simon P. Mooijaart, Karel G.M. Moons, Geeske Peeters, Harmke A. Polinder-Bos, Fenne Wouters, Anne de Hond

Table of Contents

Supplement 1: CCI score and predictors definitions with ICD-10 codes ***2***
Supplement 2: Sample size calculations ***8***
Supplement 3: Results in penalized logistic regression model ***9***
Supplement 4: Sensitivity Analysis Year 2020 .... ***10***
Supplement 5: Sensitivity analysis in year 2021 .... ***12***
Supplement 6: Participant descriptives based on level of care ***14***
Supplement 7: TRIPOD checklist ***15***

Supplement 1: CCI score and predictors definitions with ICD-10 codes

Table 1: Components of Charlson Comorbidity Index

Condition		Weight [points]
Age	70 to 79 years	3
	≥80 years	4
Myocardial infarction		1
Congestive heart failure		1
Peripheral Vascular Disease		1
CVA or TIA		1
Dementia		1
Chronic obstructive pulmonary disease		1
Connective tissue disease		1
Peptic ulcer disease		1
Liver disease	Mild	1
	Moderate to severe	3
Diabetes mellitus	uncomplicated	1
	End-organ damage	2
Hemiplegia		2
Moderate to severe chronic kidney disease		2
Solid tumor	Localized	2
	Metastatic	6
Leukaemia		2
Lymphoma		2
AIDS		6

CVA or TIA: cerebrovascular accident or transient ischemic attacks, AIDS: Acquired immunodeficiency syndrome

Table 2: Predictors needed for Charlson Comorbidity Index

Predictors	ICD-10 codes	Definition and categories	Terms used for extraction of medical conditions
**Age (years)**			The day of hospital admission for Covid-19 (24 hours)
**Myocardial infarction**	I21, I22, I25.2	History of definite or probable MI (EKG changes and/or enzyme changes)	Extracted from case report form under liver disease using terms " Hartaanval", "Acuut myocardinfarct", "Genezen myocardinfarct", "Vroeger myocardinfarct", "acuut coronair syndroom", "myocardinfarct", "myocardinfarct in de voorwand", "voorwand infarct", "onderwand infarct", "onderwand myocardinfarct", "VW-infarct", "VW infarct", "anteroseptaal infarct", "AS-infarct", "AS infarct", "inferior infarct", "niet-ST verhoogd myocardinfarct", "shock, cardiogeen", "cardiogeen", "ST-elevatie myocardinfarct", "myocardiale reperfusieschade", "Hartinfarct", "Ischaemisch myocardinfarct ", "STEMI", "Non-STEMI", "Pijn op de borst", "ischemisch hart", "Eentaks", "tweetaks", "drietakslijden", "Hartstilstand", “ACS”, “Verdenking myocardinfarct”, “Verdenking MI”.
**Congestive heart failure**	I50, I11.0, I13.0, I13.2, I25.5, I42, I43, P29.0	Exertional or paroxysmal nocturnal dyspnea and has responded to digitalis, diuretics, or afterload-reducing agents	Extracted from case report form using terms "alcoholische cardiomyopathie", "cardiomyopathie met hartdilatatie", "cardiomyopathie primair","cardiomyopathie secundair", "Congestief hartfalen", "Constrictieve cardiomyopathie NNO", "Gedilateerde cardiomyopathie", "hartdecompensatie" "Ischemische cardiomyopathie", "Reumatische carditis" ,"Reumatische hartziekte","Restrictieve cardiomyopathie", “Hartfalen”, “HFpEF”, “HFrEF”, “Decompensatio cordis”.
**Peripheral Vascular Disease**	I70, I71, I731, I73.8, I73.9, I77.1, I79.0, I79.2, K55.1, K55.8, K55.9, Z95.8, Z95.9, R02, Z99.4	Intermittent claudication or past bypass for chronic arterial insufficiency, history of gangrene or acute arterial insufficiency, or untreated thoracic or abdominal aneurysm (≥6 cm)	Extracted from case report form using terms "arteriolosclerose","arteriosclerose","arteriosclerotische vaatziekte","Aneurysma en dissectie van aorta","Aneurysma dissecans van aorta", "Aneurysma van aorta thoracalis","Aneurysma van aorta abdominalis","Aneurysma van thoracoabdominale aorta","Thrombangiitis obliterans","Acrocyanose","Erytrocyanose","Erytromelalgie","Acroparesthesie","Erytromelalgie","Perifere vaatziekte","Claudicatio intermittens","Spasme van arterie","Strictuur van arterie","Luetisch aneurysma van aorta","Diabetische perifere angiopathie","Perifere angiopathie","Mesenteriale atherosclerose","Chronische ischemische Colitis","Chronische ischemische Enteritis", "Chronische ischemische Enterocolitis","Ischemische strictuur van darm","Mesenteriale Atherosclerose","Mesenteriale vasculaire insufficiëntie","Chronische vaataandoeningen van darm","Vaataandoening van darm","Ischemischecolitis NNO","Ischemische enteritis NNO","Ischemische enterocolitis NNO", "intravasculaire prothese NEC","Status na perifere vaatplastiek NNO", “perifeer vaatlijden” “vaatlijden”.
**CVA or TIA**	G45.0-G45.2, G45.4, G45.8, G45.9, G46, I60-I69	History of a cerebrovascular accident with minor or no residua and transient ischemic attacks	Extracted from case report form using terms "Cerebro Vasculair Accident", "Herseninfarct", "Hersenbloeding", "Beroerte", "Stroke", "Transient Ischemic Attack", "Cerebrovascular Accident", "Voorbijgaande afsluiting van retinale arteriën", "Arteria-cerebri-mediasyndroom", "Arteria-cerebri-anteriorsyndroom", "Arteria-cerebri-posteriorsyndroom", a. cerebri media infarct", "a. cerebri

			anterior infarct", "a. cerebri-posterior infarct", "Vasculair syndroom van hersenstam", "Syndroom (van) Benedikt", "Syndroom (van) Claude", "Syndroom (van) Foville", "Syndroom (van) Millard-Gubler", "Syndroom (van) Wallenberg", "Syndroom (van) Weber", "Vasculair syndroom van cerebellum", "Zuiver motorisch lacunair syndroom", "Zuiver sensorisch lacunair syndroom", "Overige lacunaire syndromen", "Transient (cerebral) ischaemic attacks", "[TIA]", “CVA”, "Arteria-carotissyndroom (hemisferisch)", “a. carotissyndroom”, "Multipele precerebrale-arteriesyndromen", "bilaterale precerebrale-arteriesyndromen", "Amaurosis fugax", "Transient global amnesia", "Cerebrale arteriitis", "Occlusie van cerebrale arteriën", "stenose van cerebrale arteriën", "Occlusie van arteria cerebri media", "stenose van arteria cerebri media", "Occlusie van arteria cerebri anterior", "stenose van arteria cerebri anterior", "stenose van arteria cerebri posterior", "stenose van arteria cerebri posterior", "Occlusie van arteriae cerebellares", "stenose van arteriae cerebellares", "Occlusie van multipele cerebrale arteriën", "stenose van multipele cerebrale arteriën", "Occlusie van bilaterale cerebrale arteriën", "stenose van bilaterale cerebrale arteriën".
**Dementia**	F00-F03, F05.1, G30, G31.1	Chronic cognitive deficit	Extracted from case report form using terms "Dementia", "Alzheimer", "Dementie", "Cognitieve achteruitgang", "Mild cognitive impairment", "Cognitieve stoornis", "Anamnetische stoornis", "SDAT", "MCI", "Seniele dementie NNO", "Preseniele dementie NNO", "Preseniele psychose NNO", "Seniele dementie, depressieve of paranoïde vorm", "Dementie", "Vasculaire dementie", "Multi-infarct dementie", "Subcorticale vasculaire dementie", "Ziekte van Alzheimer", "Seniele hersendegeneratie NEC", “cognitieve stoornissen” and “cognitieve beperkingen” and “cognitieve schade” and “dementieel syndroom”, “lewybodydementie”, “LBD” , “lewy body dementia”, “parkinsondementie”, “frontotemporale dementie” ,“fronto-temporale dementie” ,“FTD”, “ziekte van Pick”.
**COPD**	J40-J47, J60-J67		Extracted from case report form using terms “Chronische Obstructieve Long Ziekte”, “Chronische bronchitis”, “Longemfyseem”, "COPD", "Chronische aspecifieke respiratoire aandoeningen "," bronchitis"," emfyseem"
**Connective tissue disease**	M05, M06.0, M06.3, M06.9, M32, M33.2, M34, M35.3		Extracted from case report form using terms "Reumatoïde artritis", "Reuscelarteriitis met polymyalgia rheumatica", "Lupus erythematodes disseminatus","LED","Dermatopolymyositis","Sclerodermie", "Systemische sclerose","Sclerodermie", "Mixed connective tissue disease", "Polymyalgia rheumatica","Dermato(poly)myositis"
**Peptic ulcer disease (Any history of treatment for ulcer disease or history of ulcer bleeding)**	K25-K28	Any history of treatment for ulcer disease or history of ulcer bleeding	Ulcus ventriculi, ulcus (pepticum) van Maag, ulcus (pepticum) van pylorus, ulcus deodeni, ulcus (pepticum) (van) Duodenum, ulcus (pepticum) (van) postpylorisch, Ulcus pepticum,ulcus gastrojejunale.

**Liver disease**	B18, K70.0-K70.3, K70.9, K71.3-K71.5, K71.7, K73, K74, K76.0, K76.2-K76.4, K76.8, K76.9, Z94.4 Severe liver disease: I85.0, I85.9, I86.4, I98.2, I98.3, K70.4, K71.1, K72.1, K72.9, K76.5, K76.6, K76.7	(Severe = cirrhosis and portal hypertension with variceal bleeding history, moderate = cirrhosis and portal hypertension but no variceal bleeding history, mild = chronic hepatitis (or cirrhosis without portal hypertension)	Mild to moderate:<br>"Chronische virushepatitis", "Chronische hepatitis B", "Chronische hepatitis C","Alcoholische vetlever", "Alcoholische hepatitis", "Alcoholische leverfibrose", "Alcoholische leversclerose", "Alcoholische levercirrose", "Alcoholische leverziekte","Toxische leverziekte","Leverfibrose", "cirrose", "cirrhose", alcoholische hepatitis cirrhose","Alcoholische hepatitis Cirrose", "levercirrose", "Vette lever","leverfalen", "lever ontsteking", "leververvetting","Portale hypertensie","Vettige leverdegeneratie", "Centrale hemorragische levernecrose", "Leverinfarct", "Peliosis hepatis","levertransplantaat","Enkelvoudige levercyste","Niet-alcoholische vetleverziekte (NAFLD/NASH)", "Hepatoptose","steatosis hepatis", "steatose",Child-Pugh<br>Ernstige leverziekte<br>Giftige leverziekte, portale hypertensie, portale biliopathie, hepatorenaal syndroom, Slokdarmvarices, Varices van maag, Alcoholische leverinsufficiëntie, Toxische leverziekte met levernecrose,Chronische leverinsufficiëntie, Veno-occlusive disease' [VOD] van lever
**Diabetes mellitus**	Diabetes without complication: E10.9, E11.9, E12.9, E13.9, E14.9 With complications: E10.0-E10.8, E11.0-E11.8, E12.0-E12.8, E13.0-E13.8, E14.0-E14.8	-None or diet-controlled<br>-Uncomplicated<br>-End-stage disease	Extracted from case report form using terms<br>Diabetes without Chronic Complications: "Diabetes", "suikerziekte", "DM", "IDDM", "diabetes mellitus", "DMII", "DMI", "Diabetes insipidus", "Type 1 diabetes mellitus", "Diabetische", "Diabetische artropathie", "Diabetische neuropathische artropathie", "Diabetisch coma", "Diabetisch hyperosmolair coma", "Diabetisch hypoglykemisch coma", "Type 2 diabetes mellitus", "Diabetische acidose".<br><br>Diabetes with Chronic Complications<br>"Diabetische complicaties", "Diabetische nefropathie", "Intracapillaire glomerulonefrose", "Syndroom van Kimmelstiel-Wilson", "Diabetisch Cataract", "Diabetisch retinopathie", "Diabetische Amyotrofie", "Diabetische autonome neuropathie", "Diabetische Mononeuropathie", "Diabetische Polyneuropathie", "Diabetische polyneuropatische autonome neuropathie", "Diabetisch Gangreen", "Diabetisch perifere angiopathie", "Diabetisch ulcus", "Diabetisch gangreen", "Diabetisch perifere angiopathie", "Diabetische amyotrofie", "Diabetische autonome neuropathie", "Diabetisch mononeuropathie", "“Diabetische neuropathie”, "Diabetische polyneuropathie", "Diabetische polyneuropatische autonome neuropathie", Diabetische nier(insufficiëntie), Diabetische proteïnurie
**Hemiplegia or paraplegia**	G04.1, G11.4, G80.1, G80.2, G81,		Extracted from case report form using terms "Tropische spastische-paraplegie", "Hereditaire spastische paraplegie", "Spastische cerebrale paralyse" , "Spastische hemiplegische cerebrale paralyse", "Spastische diplegische cerebrale paralyse", "Hemiplegie",

	G82, G83.0-G83.4, G83.8, G83.9		"Paraplegie", "Tetraplegie", "Diplegia", "Hoog Diplegia", "Diplegia van armen", "Paralyse van beide armen", "Paralyse van been", "Monoplegie van been", "Paralyse van arm","Monoplegie van arm" "Monoplegie", "Cauda-equinasyndroom","Paralytisch syndroom", “Halfzijdige verlamming”,“Volledige verlamming”,“volledige dwarsleasie”
**Moderate to severe CKD**	I12.0, I13.1, N01, N03, N05.2-N05.7, N18, N19, N25, Z49.0-Z49.2, Z94.0, Z99.2	Severe = on dialysis, status post kidney transplant, uremia<br>moderate = creatinine >3 mg/dL (0.27 mmol/L)	Extracted from case report form using terms “nierfalen” ,"Chronisch nierfalen", "Chronische nierinsufficiëntie", "Chronische nierinsufficiëntie", "Nierfalen", "Nierinsufficiëntie", "Dialyse", "Nefropathie", "Nierfunctiestoornis", "Verminderde nierfunctie", "Slechte nierfunctie", "Hypertensieve nierziekte", "Hypertensieve nierinsufficiëntie", “nierdialyse” and “peritoneaaldialyse”, “CAPD”.<br>All numeric possibilities for eGFR < 45.
**Solid tumor**	C00-C26, C30-C34, C37-C41, C43, C45-C58, C60-C76<br>Metastatic : C77-C80	None<br>Localized<br>Metastatic	Extracted from case report form using terms<br>SOLID TUMOR: "solide tumor", Hersentumor, "gelokaliseerde tumor" or "locale tumor"<br>Malignancy: "Maligniteit", "carcinoom", "Maligne neoplasma", "Maligne melanoom", "Secundair maligne neoplasma", "Choriocarcinoom", "Chorionepithelioom",“borstkanker”, “mammacarcinoom”, longkanker, darmkanker, Blaaskanker,Slokdarmkanker, Schildklierkanker, Slokdarmkanker, Galblaaskanker, Bijnierkanker,Botkanker, mammacarcinoom”, “prostaatkanker”,<br>“NSCLC”<br>Prostaatkanker<br>Nierkanker<br>Leverkanker<br>Maagkanker<br>Eierstokkanker<br>Baarmoederhalskanker<br>Pancreaskanker
**Leukemia**	C90-C97<br>D47.5		Extracted from case report form using terms "Acute leukemie", "Chronische leukemie", "Acute lymfatische leukemie", "Acute myeloïde leukemie", "Chronische lymfatische leukemie", "Chronische myeloïde leukemie", "T-cel-leukemie/lymfoom", "Chronische lymfatische B-celleukemie", "B-CLL", "CML", "CLL", "AML", "ALL", "Lymfatische leukemie", "Myeloïde leukemie", "Monocytaire leukemie", "hematologische kanker", "bloedkanker", "hemato-oncologische ziekte", “hematologische maligniteit”, “heaematologische maligniteit”
**Lymphoma**	C81-C85, C86.0-C86.6, C88		Extracted from case report form using terms "Hodgkinlymfoom", "Hodgkin lymfoom", "Non-hodgkinlymfomen", "Non-hodgkin lymfoom", "Ziekte van Hodgkin", "Maligne lymfoom", "HL", "NHL", "non-Hodgkin-lymfoom", "Hodgkin-lymfoom", "Folliculair lymfoom", "Non-folliculair lymfoom", "Lymfomen", "Maligne immunoproliferatieve ziekten", "Maligne immunoproliferatieve aandoeningen", "Maligne neoplasmata", "Multipel myeloom", "maligne neoplasmata van plasmacellen", "Multipel myeloom", "Plasmacelleukemie", "Extramedullair plasmacytoom",

		"Solitair plasmocytoom", “Lymfeklierkanker”, “waldenstrom”,"Hodgkinlymf", "Hodgkin lymf", "Non-hodgkinlymf", "Non-hodgkin lymf", "Maligne lymf", "non-Hodgkin-lymf", "Hodgkin-lymf", "Folliculair lymf", "Non-folliculair lymf",
**AIDS**	B20-B24,<br>O98.7	Extracted from case report form using terms "AIDS", "acquired immunodeficiency syndrome", "HIV", "Humaan Immunodeficiency Virus", "Humaan Immunodeficiëntievirus [HIV]", "AIDS-related complex", "Acquired immunodeficiency syndrome [AIDS]", "Ziekte door Humaan Immunodeficiëntievirus", "HIV-ziekte", “hiv/aids” and “hiv-positief”.<br>Exclusie: "HIV negatief", "HIV neg", “Hiv sneltest neg”.

Supplement 2: Sample size calculations

If we assume a c-statistic of 0.7, a mortality rate of 0.185 (based on previous studies in nursing homes), and 10 or 13 candidate predictor terms, we will need at least 1137 participants to have robust models with a maximum shrinkage of 10% (pmsampsizepackage). Mortality fraction was based on a previous study with a mortality fraction of 18.5% from March 2020 to December 2021 (6). Based on the different number of predictor parameters, the sample size calculations can be visualized in Table 2.

Table 2: Required sample size based on pmsampsize, with shrinkage = 0.9

Parameters	Mortality fraction	C-statistic	Required sample size	Events per predictor
**10**	0.185	0.70	1137	21.03
**10**	0.185	0.65	2073	38.35
**10**	0.185	0.60	4763	88.12
**13**	0.185	0.70	1478	21.03
**13**	0.185	0.65	2694	38.34
**13**	0.185	0.60	6192	88.12

Supplement 3: Results in penalized logistic regression model

Table 1: Results of penalized models

	Base model<br>(age + sex + age*sex)	CCI model<br>Base model + CCI
**AUROC, (95% CI)**	0.61 (0.60 – 0.61)	0.62 (0.60-0.63)
**Scaled Brier score, (95% CI)**	0.04 (0.03 to 0.05)	0.04 (0.03 to 0.05)
**Calibration slope, (95% CI)**	1.00 (0.84 to 1.17)	1.01 (0.84 to 1.17)

Table 2: Range of predicted risks in base and CCI models

	Predicted risks				
	**Minimum**	**25%**	**Median**	**75%**	**Maximum**
**Base model**	0.129	0.254	0.314	0.377	0.636
**CCI model**	0.113	0.249	0.311	0.382	0.664

Supplement 4: Sensitivity Analysis Year 2020

Table 1: Characteristics of study participants stratified on mortality status in year 2020

Characteristics		All participants (n=1718)	Survivors (n=1019)	28-day mortality (n=699)
**Age (years), median [IQR]**		89 [84-94]	89 [83-94]	89 [84-94]
**Male, n (%)**		588 (34)	286 (28)	302 (43)
**Mortality, n (%)**		699 (41)	-	-
**Charlson comorbidity index, median [IQR]**		6 [5-7]	6 [5-7]	6 [5-7]
**Dementia, n (%)**		1251 (73)	740 (73)	511 (73)
**COPD, n (%)**		106 (6)	66 (6)	40 (6)
**Diabetes**	**Uncomplicated, n (%)**	346 (20)	202 (20)	144 (21)
	**End-organ damage, n (%)**	21 (1)	12 (1)	9 (1)
**Chronic kidney disease, n (%)**		304 (18)	164 (16)	140 (20)
**Liver disease**	**Mild n (%)**	3 (<1)	3 (<1)	1 (0)
	**Severe n (%)**	26 (2)	17 (2)	9 (1)
**Myocardial infarction, n (%)**		159 (9)	91 (9)	68 (10)
**Chronic heart failure, n (%)**		276 (16)	158 (16)	118 (17)
**Peripheral vascular disease, n (%)**		58 (3)	32 (3)	26 (4)
**Cerebrovascular accident, n (%)**		228 (13)	134 (13)	94 (13)
**Connective tissue disease, n (%)**		224 (13)	160 (16)	64 (9)
**Peptic ulcer disease, n (%)**		41 (2)	27 (3)	14 (2)
**Paralysis, n (%)**		5 (<1)	0 (0)	5 (<1)
**Solid tumor**	**Localized, n (%)**	370 (22)	235 (23)	135 (19)
	**Malignant, n (%)**	31 (2)	19 (1)	12 (2)
**Leukemia, n (%)**		8 (<1)	6 (<1)	2 (<1)
**Lymphoma, n (%)**		7 (<1)	6 (1)	1 (<1)

Table 2: Range of predicted risks in base and CCI models

	Predicted risks				
Model	**Minimum**	**25%**	**Median**	**75%**	**Maximum**
Base model	0.224	0.344	0.379	0.510	0.606
Base model + CCI	0.196	0.340	0.380	0.492	0.634

Table 3: Results of penalized model performance in year 2020

**Model**	AUROC	Scaled brier score	Calibration slope
**Base model**	0.61 (0.58-0.63)	0.04 (0.02-0.06)	1.01 (0.76-1.26)
**Base model + CCI**	0.61 (0.59-0.64)	0.04 (0.03-0.07)	1.01 (0.77-1.25)

Table 4: Results of optimism corrected model performance using bootstrapping (n=1000) in year 2020

**Model**	**Optimism corrected AUC**	**Optimism corrected Brier scaled**	**Optimism corrected Calibration slope**
**Base model**	0.60 (0.57-0.63)	0.03 (0.01-0.05)	0.93 (0.74-1.20)
**Base model + CCI**	0.60 (0.58-0.63)	0.03 (0.01-0.05)	0.89 (0.72-1.13)

Figure 1: Distributions of predicted risks by penalized models stratified by outcome. Plot A shows the 28-day mortality risks as predicted by the base model, whereas plot B shows the risks predicted for CCI model (both models showed very similar distribution). For each plot, the distribution of predicted risks for patients who survived 28 days is shown in blue, and the distribution of predicted risks for patients who died within 28 days is shown in yellow.

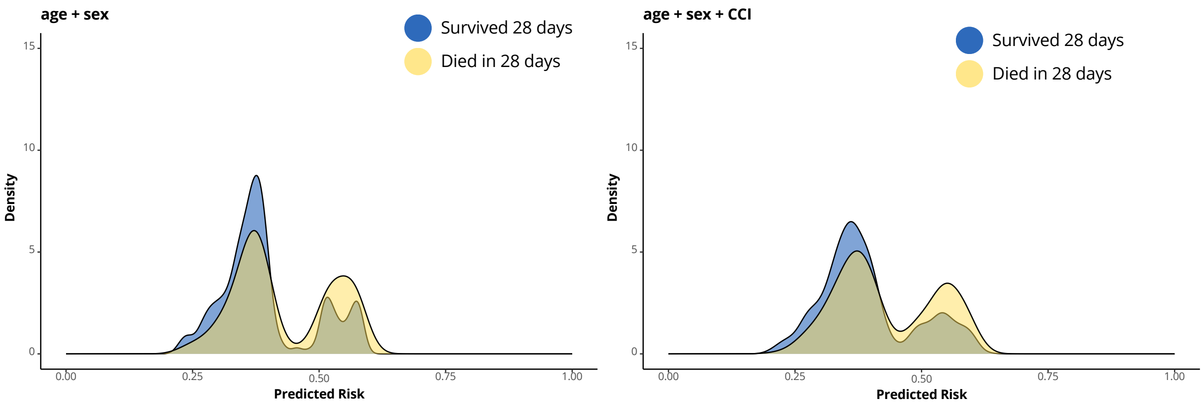

CCI = Charlson Comorbidity Index

Supplement 5: Sensitivity analysis in year 2021

Table 1: Characteristics of study participants stratified on mortality status in year 2021

Characteristics		All participants (n=2600)	Survivors (n=1942)	28-day mortality (n=658)
**Age (years), median [IQR]**		87 [82-93]	87 [81-92]	89 [84-94]
**Male, n (%)**		938 (36)	645 (33)	293 (45)
**Mortality, n (%)**		658 (25)	-	-
**Charlson comorbidity index, median [IQR]**		6 [5-7]	6 [5-7]	6 [5-8]
**Dementia, n (%)**		1630 (63)	1225 (63)	405 (62)
**COPD, n (%)**		170 (7)	124 (6)	46 (7)
**Diabetes**	**Uncomplicated, n (%)**	549 (21)	415 (21)	134 (20)
	**End-organ damage, n (%)**	22 (1)	15 (1)	7 (1)
**Chronic kidney disease, n (%)**		418 (16)	287 (15)	131 (20)
**Liver disease**	**Mild, n (%)**	5 (<1)	4 (<1)	1 (<1)
	**Severe, n (%)**	34 (1)	25 (1)	9 (1)
**Myocardial infarction, n (%)**		256 (10)	190 (10)	66 (10)
**Chronic heart failure, n (%)**		441 (17)	308 (16)	133 (20)
**Peripheral vascular disease, n (%)**		123 (5)	93 (5)	30 (5)
**Cerebrovascular accident, n (%)**		338 (13)	242 (12)	96 (15)
**Connective tissue disease, n (%)**		395 (15)	318 (16)	77 (12)
**Peptic ulcer disease, n (%)**		45 (2)	29 (1)	16 (2)
**Paralysis, n (%)**		7 (<1)	7 (<1)	0 (0)
**Solid tumour**	**Localized, n (%)**	602 (23)	441 (23)	161 (24)
	**Malignant, n (%)**	56 (2)	41 (2)	15 (2)
**Leukaemia, n (%)**		7 (<1)	3 (1)	4 (1)
**Lymphoma, n (%)**		15 (1)	10 (1)	5 (1)

Table 2: Range of predicted risks in base and CCI models

			Predicted risks		
**Model**	**Minimum**	**25%**	**Median**	**75%**	**Maximum**
**Base model**	0.08	0.20	0.26	0.30	0.64
**Base model + CCI**	0.08	0.19	0.26	0.30	0.68

Table 3: Results of penalized model performance in year 2021

Model	AUROC	Scaled brier score	Calibration slope
**Base model**	0.62 (0.60-0.65)	0.04 (0.03-0.06)	1.01 (0.80-1.22)
**Base model + CCI**	0.62 (0.60-0.65)	0.04 (0.03-0.06)	1.01 (0.80-1.22)

Table 4: Results of optimism corrected model performance using bootstrapping (n=1000) in year 2021

Model	Optimism corrected AUC	Optimism corrected Brier scaled	Optimism corrected Calibration slope
**Base model**	0.62 (0.60-0.64)	0.03 (0.01-0.04)	0.94 (0.77-1.16)
**Base model + CCI**	0.61 (0.59-0.64)	0.03 (0.01-0.04)	0.92 (0.75-1.12)

Figure 1: Distributions of predicted risks by penalized models stratified by outcome. Plot A shows the 28-day mortality risks as predicted by the base model, whereas plot B shows the risks predicted for CCI model (both models showed very similar distribution). For each plot, the distribution of predicted risks for patients who survived 28 days is shown in blue, and the distribution of predicted risks for patients who died within 28 days is shown in yellow.

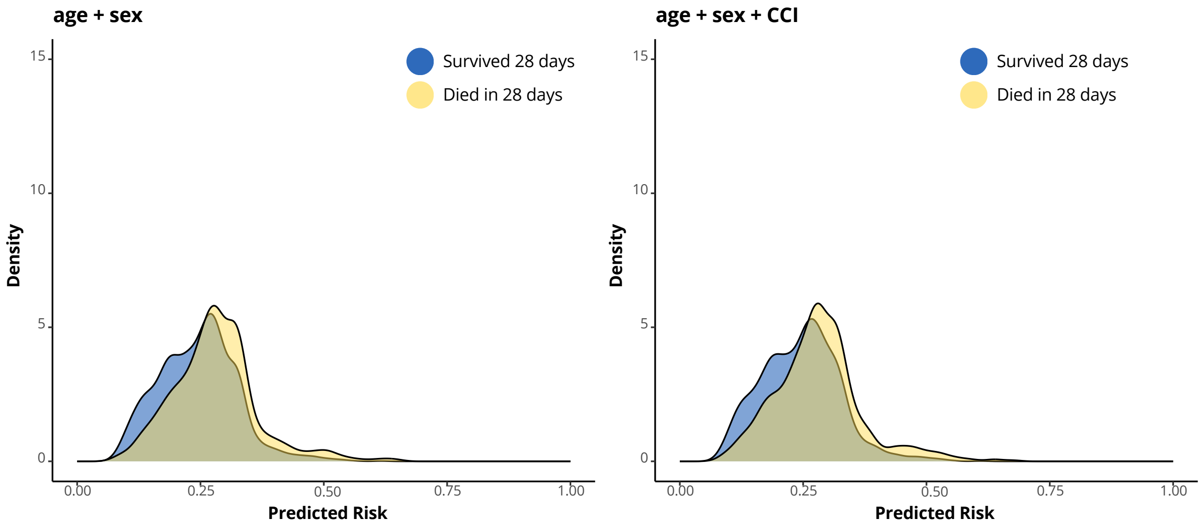

Supplement 6: Participant descriptives based on level of care

Table 1: Characteristics of study participants stratified by level of care

Characteristics		Chronic Psychogeriatric care (n=2447)	Chronic somatic care (n=1030)	Short-term care (Revalidation) (n=841)
**Age (years), median [IQR]**		88 [83-93]	88 [82-94]	87 [81-92]
**Male, n (%)**		824 (34)	360 (35)	342 (41)
**Mortality, n (%)**		784 (32)	291 (28)	282 (34)
**Charlson comorbidity index, median [IQR]**		6 [5 - 7]	6 [5 - 8]	6 [5 - 7]
**Dementia, n (%)**		2253 (92)	315 (31)	313 (37)
**COPD, n (%)**		127 (5)	80 (8)	69 (8)
**Diabetes, n (%)**	**Uncomplicated**	465 (19)	248 (24)	182 (2)
	**End-organ damage**	15 (1)	19 (2)	9 (1)
**Chronic kidney disease, n (%)**		369 (15)	201 (20)	152 (18)
**Liver disease, n (%)**	**Mild**	1 (<1)	4 (<1)	3 (<1)
	**Severe**	32 (1)	19 (2)	9 (1)
**Myocardial infarction, n (%)**		206 (8)	120 (12)	89 (11)
**Chronic heart failure, n (%)**		296 (12)	253 (25)	168 (20)
**Peripheral vascular disease, n (%)**		79 (3)	57 (6)	45 (5)
**Cerebrovascular accident, n (%)**		230 (9)	211 (20)	125 (15)
**Connective tissue disease, n (%)**		332 (14)	168 (16)	119 (14)
**Peptic ulcer disease, n (%)**		48 (2)	27 (3)	11 (1)
**Paralysis, n (%)**		0 (0)	7 (1)	5 (1)
**Solid tumor, n (%)**	**Localized**	527 (22)	259 (25)	186 (22)
	**Malignant**	32 (1)	30 (3)	25 (3)
**Leukemia, n (%)**		3 (<1)	6 (1)	6 (1)
**Lymphoma, n (%)**		10 (<1)	8 (1)	4 (<1)

Supplement 7: TRIPOD checklist

TRIPOD+AI

Version: 11-January-2024

Section/Topic	Item	Development / evaluation[1]	Checklist item	Reported on page
**TITLE**				
Title	1	D;E	Identify the study as developing or evaluating the performance of a multivariable prediction model, the target population, and the outcome to be predicted	1
**ABSTRACT**				
Abstract	2	D;E	See TRIPOD+AI for Abstracts checklist	
**INTRODUCTION**				
Background	3a	D;E	Explain the healthcare context (including whether diagnostic or prognostic) and rationale for developing or evaluating the prediction model, including references to existing models	5
	3b	D;E	Describe the target population and the intended purpose of the prediction model in the context of the care pathway, including its intended users (e.g., healthcare professionals, patients, public)	5
	3c	D;E	Describe any known health inequalities between sociodemographic groups	NA
Objectives	4	D;E	Specify the study objectives, including whether the study describes the development or validation of a prediction model (or both)	5
**METHODS**				
Data	5a	D;E	Describe the sources of data separately for the development and evaluation datasets (e.g., randomised trial, cohort, routine care or registry data), the rationale for using these data, and representativeness of the data	6
	5b	D;E	Specify the dates of the collected participant data, including start and end of participant accrual; and, if applicable, end of follow-up	6
Participants	6a	D;E	Specify key elements of the study setting (e.g., primary care, secondary care, general population) including the number and location of centres	6
	6b	D;E	Describe the eligibility criteria for study participants	6
	6c	D;E	Give details of any treatments received, and how they were handled during model development or evaluation, if relevant	NA
Data preparation	7	D;E	Describe any data pre-processing and quality checking, including whether this was similar across relevant sociodemographic groups	NA
Outcome	8a	D;E	Clearly define the outcome that is being predicted and the time horizon, including how and when assessed, the rationale for choosing this outcome, and whether the method of outcome assessment is consistent across sociodemographic groups	6
	8b	D;E	If outcome assessment requires subjective interpretation, describe the qualifications and demographic characteristics of the outcome assessors	NA
	8c	D;E	Report any actions to blind assessment of the outcome to be predicted	NA
Predictors	9a	D	Describe the choice of initial predictors (e.g., literature, previous models, all available predictors) and any pre-selection of predictors before model building	6
	9b	D;E	Clearly define all predictors, including how and when they were measured (and any actions to blind assessment of predictors for the outcome and other predictors)	Supplement 1
	9c	D;E	If predictor measurement requires subjective interpretation, describe the qualifications and demographic characteristics of the predictor assessors	NA
Sample size	10	D;E	Explain how the study size was arrived at (separately for development and evaluation), and justify that the study size was sufficient to answer the research question. Include details of any sample size calculation	Supplement 2
Missing data	11	D;E	Describe how missing data were handled. Provide reasons for omitting any data	5
Analytical methods	12a	D	Describe how the data were used (e.g., for development and evaluation of model performance) in the analysis, including whether the data were partitioned, considering any sample size requirements	7
	12b	D	Depending on the type of model, describe how predictors were handled in the analyses (functional form, rescaling, transformation, or any standardisation).	7
	12c	D	Specify the type of model, rationale[2], all model-building steps, including any hyperparameter tuning, and method for internal validation	6-8
	12d	D;E	Describe if and how any heterogeneity in estimates of model parameter values and model performance was handled and quantified across clusters (e.g., hospitals, countries). See TRIPOD-Cluster for additional considerations[3]	NA
	12e	D;E	Specify all measures and plots used (and their rationale) to evaluate model performance (e.g., discrimination, calibration, clinical utility) and, if relevant, to compare multiple models	6-8
	12f	E	Describe any model updating (e.g., recalibration) arising from the model evaluation, either overall or for particular sociodemographic groups or settings	NA
	12g	E	For model evaluation, describe how the model predictions were calculated (e.g., formula, code, object, application programming interface)	6-8
Class imbalance	13	D;E	If class imbalance methods were used, state why and how this was done, and any subsequent methods to recalibrate the model or the model predictions	NA
Fairness	14	D;E	Describe any approaches that were used to address model fairness and their rationale	NA
Model output	15	D	Specify the output of the prediction model (e.g., probabilities, classification). Provide details and rationale for any classification and how the thresholds were identified	7-8

[1] D=items relevant only to the development of a prediction model; E=items relating solely to the evaluation of a prediction model; D;E=items applicable to both the development and evaluation of a prediction model

[2] Separately for all model building approaches.

[3] TRIPOD-Cluster is a checklist of reporting recommendations for studies developing or validating models that explicitly account for clustering or explore heterogeneity in model performance (eg, at different hospitals or centres). Debray et al, BMJ 2023; 380: e071018 [DOI: 10.1136/bmj-2022-071018]

Training versus evaluation	16	D;E	Identify any differences between the development and evaluation data in healthcare setting, eligibility criteria, outcome, and predictors	NA
Ethical approval	17	D;E	Name the institutional research board or ethics committee that approved the study and describe the participant-informed consent or the ethics committee waiver of informed consent	11
**OPEN SCIENCE**				
Funding	18a	D;E	Give the source of funding and the role of the funders for the present study	12
Conflicts of interest	18b	D;E	Declare any conflicts of interest and financial disclosures for all authors	11
Protocol	18c	D;E	Indicate where the study protocol can be accessed or state that a protocol was not prepared	Not published
Registration	18d	D;E	Provide registration information for the study, including register name and registration number, or state that the study was not registered	NA
Data sharing	18e	D;E	Provide details of the availability of the study data	11
Code sharing	18f	D;E	Provide details of the availability of the analytical code[4]	NA
**PATIENT & PUBLIC INVOLVEMENT**				
Patient & Public Involvement	19	D;E	Provide details of any patient and public involvement during the design, conduct, reporting, interpretation, or dissemination of the study or state no involvement.	12
**RESULTS**				
Participants	20a	D;E	Describe the flow of participants through the study, including the number of participants with and without the outcome and, if applicable, a summary of the follow-up time. A diagram may be helpful.	7, Table 2
	20b	D;E	Report the characteristics overall and, where applicable, for each data source or setting, including the key dates, key predictors (including demographics), treatments received, sample size, number of outcome events, follow-up time, and amount of missing data. A table may be helpful. Report any differences across key demographic groups.	7-8 Table 2
	20c	E	For model evaluation, show a comparison with the development data of the distribution of important predictors (demographics, predictors, and outcome).	NA
Model development	21	D;E	Specify the number of participants and outcome events in each analysis (e.g., for model development, hyperparameter tuning, model evaluation)	7, table 2
Model specification	22	D	Provide details of the full prediction model (e.g., formula, code, object, application programming interface) to allow predictions in new individuals and to enable third-party evaluation and implementation, including any restrictions to access or re-use (e.g., freely available, proprietary)[5]	NA
Model performance	23a	D;E	Report model performance estimates with confidence intervals, including for any key subgroups (e.g., sociodemographic). Consider plots to aid presentation.	8, Table 3, Figure 2
	23b	D;E	If examined, report results of any heterogeneity in model performance across clusters. See TRIPOD Cluster for additional details[3].	NA
Model updating	24	E	Report the results from any model updating, including the updated model and subsequent performance	NA
**DISCUSSION**				
Interpretation	25	D;E	Give an overall interpretation of the main results, including issues of fairness in the context of the objectives and previous studies	10
Limitations	26	D;E	Discuss any limitations of the study (such as a non-representative sample, sample size, overfitting, missing data) and their effects on any biases, statistical uncertainty, and generalizability	9-10
Usability of the model in the context of current care	27a	D	Describe how poor quality or unavailable input data (e.g., predictor values) should be assessed and handled when implementing the prediction model	NA
	27b	D	Specify whether users will be required to interact in the handling of the input data or use of the model, and what level of expertise is required of users	NA
	27c	D;E	Discuss any next steps for future research, with a specific view to applicability and generalizability of the model	11

From: Collins GS, Moons KGM, Dhiman P, et al. *BMJ* 2024;385:e078378. doi:10.1136/bmj-2023-078378
